# Involvement of SLC39A6 in gastric adenocarcinoma and correlation of the SLC39A6 polymorphism rs1050631 with clinical outcomes after resection

**DOI:** 10.1186/s12885-019-6222-z

**Published:** 2019-11-08

**Authors:** Jian Gao, Wenjun Ren, Chunhong Xiao, Lie Wang, Qiaojia Huang, Zaizhong Zhang, Yuan Dang, Pengcheng Weng, Hui Wang, Xuehong Fang, Minxian Zhuang, Liying Lin, Shaoquan Chen

**Affiliations:** 1 Department of General Surgery, Dongfang Hospital (900 Hospital of the Joint Logistics Team), 156 North Xi-er Huan Road, Fuzhou, 350025 Fujian China; 2 China Clinical Institute of Fuzhou General Hospital (900 Hospital of the Joint Logistics Team), Fujian Medical University, 156 North Xi-er Huan Road, Fuzhou, 350025 Fujian China; 3 Department of Experimental Medicine, Dongfang Hospital (900 Hospital of the Joint Logistics Team), 156 North Xi-er Huan Road, Fuzhou, 350025 Fujian China; 4 Fujian Meiya Aijiankang Health Management Co, Ltd. 4602#, Building 1, Shimao International Center, 108 Guangda Road, Fuzhou, 350025 Fujian China; 5 Union Medical College, Fujian Medical University, 29 XinQuan Road, Fuzhou, 350025 Fujian China

**Keywords:** *SLC39A6* rs1050631, Gastric adenocarcinoma, Prognostic biomarker, High-occurrence area, Ki67, TOPOII

## Abstract

**Background:**

The single-nucleotide polymorphism SLC39A6 rs1050631 is strongly implicated in esophageal squamous cell carcinoma, leading us to question whether it may also play a role in gastric adenocarcima (GA).

**Methods:**

We genotyped the SLC39A6 rs1050631 in 512 patients who underwent GA resection. All study subjects lived in an area of China with high GA incidence. Genotypes were examined for possible correlation with survival and recurrence. The potential involvement of SLC39A6 in gastric cancer was explored in clinical samples and cell culture studies.

**Results:**

Multivariable analysis showed that patients with the CT + TT genotype at SLC39A6 rs1050631 were at greater risk of recurrence (hazard ratio, HR 1.387, *p* = 0.004) and death (HR 1.429, *p* = 0.002) than patients with CC genotype. Median recurrence-free and overall survival were significantly shorter in patients with the CT + TT genotype (20, 27 months) than in patients with the CC genotype (36, 43 months, *p* = 0.001, *p* < 0.001). Patients with the CT + TT genotype who were male or ≥ 60 years, or who had a tumor ≥5 cm or a moderately differentiated tumor were at significantly higher risk of recurrence and death. SLC39A6 was overexpressed in tissues from GA patients and in GA cell lines, and SLC39A6 knockdown in GA cell lines inhibited their proliferation, migration and invasion.

**Conclusion:**

SLC39A6 rs1050631 correlates with post-resection prognosis of GA patients and SLC39A6 may participate in GA onset or progression.

## Background

Gastric cancer is one of the most common causes of cancer-related deaths worldwide [1]. Most gastric cancer cases occur in Asia, particularly in China [2, 3]. The incidence of gastric cancer, its progression and patient prognosis differ across geographic regions and ethnic groups, and the reasons for these variations are poorly understood. A high-salt diet may stimulate gastric mucosa excessively, leading to chronic gastric inflammation and gastric carcinogenesis [4]. Consumption of *N*-nitroso-containing foods may contribute to gastric cancer, reflecting the fact that the N-nitroso group is a well-known carcinogen [5]. Genetic factors also appear to contribute to gastric cancer development and progression [6–8]. To clarify the factors contributing to gastric cancer, it may be beneficial to study populations living in areas with a high incidence of this disease, since such groups may have unique genetic backgrounds linked to the molecular mechanisms behind the illness. Therefore we examined patients who underwent surgical resection to treat gastric adenocarcinoma (GA) in the Chinese province of Fujian, one of the areas with the highest incidence of gastric cancer in China [9, 10].

We obtained clinical samples from patients in Fujian province following resection to treat GA, and examined potential association between *SLC39A6* r1050631 with clinical outcomes. We also examined the relationship between *SLC39A6* expression and *SLC39A6* r1050631. Potential effects of knocking down *SLC39A6* expression were examined in representative GA cell lines.

## Methods

### Patients

This retrospective study included 512 Han Chinese patients living in Fujian, China. Briefly, we examined whether polymorphism in the gene encoding solute carrier family 39 (zinc transporter) member 6, often referred to as SLC39A6 or LIV-1, is associated with GA. This gene is known to promote the development and metastasis of several human cancers [11, 12]. Studies involving patients from different parts of China have generated strong evidence linking *SLC39A6* overexpression with risk of esophageal squamous cell carcinoma (ESCC) and poor survival [13, 14], and linking the single-nucleotide polymorphism *SLC39A6* rs1050631 with survival [14]. The esophagus is connected physically and functionally to the stomach, yet we are unaware of studies exploring a potential link between *SLC39A6* rs1050631 and gastric cancer. Therefore we decided to focus on this polymorphism, although other polymorphisms may also be important in gastric cancer.All patients were diagnosed with primary GA. Surgical resection of the primary gastric tumors was performed between July 2003 and December 2009 at 900 Hospital of the Joint Logistics Team (Fujian, China). Pathologists confirmed the diagnosis of GA following histopathological examination of the tumor tissues. All patients had complete medical records, including detailed clinical pathological features. Recurrence was defined based on our previously described method [15]. Survival was defined as the interval from the date of surgery to the date of death or the last follow-up (November 2014). Survival information was obtained primarily through telephone interview and the Social Security Death Index system. None of the patients included into this study had received preoperative chemotherapy. Of the 512 patients, 329 received postoperative chemotherapy with epirubicin, cisplatin, fluorouracil, or one or two of these three drugs plus the remaining one or two drugs. The following data were extracted from medical records in the hospital database: age, sex, tumor differentiation grade, tumor size, tumor-node-metastasis (TNM) stage, lymph node metastasis, distant metastasis, chemotherapy status, and other clinicopathological information. TNM staging and histologic classification were performed by experienced pathologists as described [16].

### Immunohistochemical detection

SLC39A6 expression was examined in a subset of 198 randomly selected GA tissue blocks and 83 non-cancerous gastric tissues using standard immunohistochemical method. The anti-SLC39A6 antibody was from Abcam (Cambridge, MA). Immunostaining was assessed as described [16, 17]. Tissues showing scores of ≥1+ for SLC39A6 staining were defined as positive; scores of ≥2+ were defined as high expression and < 2+ as low expression.

### SNP selection and genotyping

*SLC39A6* rs1050631 was selected as the focus of the present study because of the strong evidence linking this gene to proliferation and invasion of ESCC cells, and this polymorphism to survival outcomes in ESCC patients, based on analysis of different groups of individuals from different parts of China [13, 14]. The esophagus and stomach are physically and functionally connected in the digestive tract, and *SLC39A6* rs1050631 has never been investigated in GA. Genomic DNA was extracted from 512 GA tissue samples using a QIAamp DNA FFPE Tissue Kit (Qiagen GmbH). The tissue samples had been formalin-fixed and paraffin-embedded immediately after surgical resection. Genotyping and analysis of the single-nucleotide polymorphism were performed as described [15–17]. The assay involved PCR to amplify the DNA, PCR product extension using a single primer, and product identification using MassARRAY SpectroCHIP and matrix-assisted laser desorption/ionization–time-of-flight mass spectrometry (Sequenom). Data were analyzed using TYPER software (Sequenom) [15–17].

### Cell culture and siRNA transfection

One normal gastric cell line (GES-1) and four GA cell lines (BGC-823, SGC-7901, AGS and MGC-803) were used in this investigation. The lines GES-1, BGC-823, and AGS were obtained and cultured as described. The lines SGC-7901 and MGC-803 were purchased from the Cell Bank of the Shanghai Institute of Biochemistry and Cell Biology (Chinese Academy of Sciences, Shanghai, China) and cultured in the same way as BGC-823. Two siRNAs targeting different sites in the *SLC39A6* gene were designed and synthesized by GenePharma (Shanghai, China). siRNA1 targeted nt 1213–1231 in the SLC39A6 cDNA (sense: 5′ –GCGAGGAAGUUAUCUGUAATT–3′; anti-sense: 5′–UUACAGAUAACUUCCUCGCTT–3′). siRNA2 targeted nt 658–676 in the SLC39A6 cDNA (sense: 5′ –GCAAUUUCCACACGGCAAUTT–3′; anti-sense: 5′–AUUGCCGUGUGGAAAUUGCTT–3′). A scrambled siRNA (sense: 5′–UUCUCCGAACGUGUCACGUTT–3′; anti-sense: 5′–ACGUGACACGUUCGGAGAATT–3′) was used as the negative control (NC). siRNAs were transfected into BGC-823 and SGC-7901 cells at a final concentration of 100 nM using Lipofectamine 2000.

### Quantitative RT-PCR assay

Relative levels of SLC39A6 mRNA in GES-1 cells and four GA cell lines (AGS, BGC-823, SGC-7901 and MGC-803) were determined by quantitative RT-PCR as described for lncRNA HNF-AS1 [18], with the following primers: SLC39A6, 5′-GCCTGCAGTCTTGGAAGAAG-3′ and 5′-GCCGAGTGTATCGTGGAAAT-3′; GAPDH, 5′-GGGAGCCAAAAGGGTCA-3′ and 5′-GAGTCCTTCCACGATACCAA-3 ′.

### Cell proliferation

BGC-823 and SGC-7901 cells were transfected for 48 h with anti-SLC39A6 siRNA, then 2000 cells in 100 μl medium were seeded into each well of a 96-well plate. At 2 h (0 d), 1 d, 3 d and 5 d after seeding, CCK-8 solution (10 μl per well; Boster, Wuhan, China) was placed into each well and cells were incubated at 37 °C for 1 h. Absorbance at 450 nm was measured using a SpectraMax microplate reader (Molecular Devices, Thermo Fisher, Waltham, MA, USA). Culture dish containing no cells and only medium was used as the blank control. BGC-823 and SGC-7901 proliferation were plotted with time based on absorbance at 450 nm.

### Cell migration and invasion

BGC-823 and SGC-7901 cell migration and invasion were examined almost as described [19], except that we performed measurements at 36 h and 48 h, respectively.

### Statistical analysis

Statistical analysis was performed using SPSS 17.0 (IBM, Chicago, IL, USA). All statistical tests were two-sided, and *p* < 0.05 was considered significant. The chi-square test was used to identify associations of *SLC39A6* rs1050631 genotypes with clinico-demographic characteristics, tumor pathology and outcomes (recurrence and survival). The variables used in uni- and multivariable Cox rgression were age, sex, tumor size, differentiation grade, gross findings, lymph node metastasis, distant metastasis, and chemotherapy regimen. Only sex, age and common variants or those with *p* < 0.2 in chi-square testing were adjusted in subsequent multivariable Cox models. Risks are presented as hazard ratios (HRs) and 95% confidence intervals (CIs). Chi-square partitioning was used to assess the significance of relationships between *SLC39A6* rs1050631 genotypes and survival or recurrence. Log-rank testing of Kaplan–Meier curves was used to assess associations of genotypes with recurrence-free and overall survival. Relationships among *SLC39A6* rs1050631 genotypes and recurrence/survival were assessed using multivariable Cox models that adjusted for age, sex, and lymph node metastasis status. Stratification was also performed based on certain clinico-demographic characteristics in an effort to identify factors that strongly influenced recurrence or survival; Cox models in stratified analyses did not adjust for the variable that was being tested. Two-way ANOVA was used to evaluate differences in cell proliferation among different treatment groups. One-way ANOVA and Dunnett’s multiple comparison test was used to assess the significance of differences in cell expression level, cell migration and invasion among different groups.

## Results

### Associations between SLC39A6 rs1050631 genotype and clinico-demographic characteristics

Among the 512 tissue samples from patients resected for GA, 3.9% (20 cases), 27.1% (139 cases), and 69.0% (353 cases) had the TT, CT, or CC genotype, respectively (Table 1). Genotype frequencies did not differ significantly (based on the chi-squared test) with age (< 60 vs. ≥60 years), degree of differentiation, gross findings (apophysis vs. invasion), Lauren classification (intestinal type, diffuse type, uncertain type), tumor location (upper third, middle third, lower third), tumor size (≥5 vs. < 5 cm), distant metastasis status or chemotherapy status (Table 1). Potential association was observed between genotype and lymph node metastasis status (*p* < 0.2). Genotype showed a significant association with post-resection survival (*p* = 0.002) and recurrence (*p* = 0.004) (Table 1).

**Table 1:** Patient characteristics and distribution of *SLC39A6* rs1050631 genotypes

Variables	Patients N (%)	SLC39A6 rs1050631 genotype (N)			P^*^	*P*_dominant_^*a*^	*P*_additive_^*b*^
		TT(%)	CT(%)	CC(%)			
Total	512	20 (3.9)	139 (27.1)	353 (69.0)			
Age					0.26	0.25	0.14
< 60	235 (45.9)	6 (1.2)	61 (11.9)	168 (32.8)			
≥ 60	277 (54.1)	14 (2.7)	78 (15.2)	185 (36.1)			
Gender					0.38	0.18	0.25
Male	383 (74.8)	15 (2.9)	110 (21.5)	258 (50.4)			
Female	129 (25.2)	5 (1.0)	29 (5.7)	95 (18.5)			
Grade of differentiation					0.85	0.93	0.86
Well differentiated	21 (4.1)	1 (0.2)	6 (1.2)	14 (2.7)			
Moderately differentiated	263 (51.4)	9 (1.8)	75 (14.6)	179 (35.0)			
Poorly differentiated	228 (44.5)	10 (2.0)	58 (11.3)	160 (31.2)			
Gross findings					0.79	0.96	0.81
Apophysis	35 (6.8)	2 (0.4)	9 (1.8)	24 (4.7)			
Invasion	477 (93.2)	18 (3.5)	130 (25.4)	329 (64.3)			
Lauren classification					0.65	0.45	0.22
Intestinal type	420 (82.1)	18 (3.5)	119 (23.2)	283 (55.3)			
Diffuse type	81 (15.8)	2 (0.4)	18 (3.5)	61((11.9))			
Uncertain type	11 (2.1)	0 (0)	2 (0.4)	9 (1.8)			
Tumor location					0.32	0.38	0.42
Upper third	179 (35.0)	4 (0.8)	52 (10.2)	123 (24.0)			
Middle third	78 (15.2)	4 (0.8)	16 (3.1)	58 (11.3)			
Lower third	252 (49.2)	12 (2.3)	69 (13.5)	171 (33.4)			
Total	3 (0.6)	0 (0)	2 (0.4)	1 (0.2)			
Tumor size					0.99	0.88	0.92
≥ 5 cm	210 (41.0)	8 (1.6)	58 (11.3)	144 (28.1)			
< 5 cm	302 (59.0)	12 (2.3)	81 (15.8)	209 (40.8)			
Survival					0.002	< 0.001	< 0.001
Alive	182 (35.5)	4 (0.4)	35 (6.8)	143 (27.9)			
Dead	330 (64.5)	16 (3.1)	104 (20.3)	210 (41.0)			
Recurrence					0.004	< 0.001	< 0.001
Yes	334 (65.2)	16 (3.1)	104 (20.3)	214 (41.8)			
No	178 (34.8)	4 (0.8)	35 (6.8)	139 (27.1)			
Distant metastasis					0.43	0.87	0.81
Yes	63 (12.3)	4 (0.8)	15 (2.9)	44 (8.6)			
No	449 (87.7)	16 (3.1)	124 (24.2)	309 (60.4)			
Lymph node metastasis					0.12	0.21	0.08
Yes	398 (77.7)	19 (3.7)	110 (21.5)	269 (52.5)			
No	114 (22.3)	1 (0.2)	29 (5.7)	84 (16.4)			
Chemotherapy							
Yes	329 (64.3)	13 (2.5)	93 (18.2)	223 (43.6)	0.61	0.78	0.69
No	183 (35.7)	7 (1.4)	46 (9.0)	130 (25.4)			

^*^ The χ^2^ test was used to calculate p values. Fisher’s exact test was used in the analysis of contingency tables when sample size was smaller than 5

^a^ Logistic regression analysis for the dominant model (TT + CT vs CC) was used to calculate the *p* value

^b^ Logistic regression analysis for the log-additive model was used to calculate the *p* value by comparing the effect of each additional variant allele

### Associations between SLC39A6 rs1050631 genotype and GA recurrence

Of all 512 patients, 334 (65.2%) experienced recurrence. Of the 353 patients with the CC genotype, 214 (60.6%) experienced recurrence (Tables 1 and 2). A significantly higher proportion of patients with the CT (104 of 139, 74.8%) or TT genotype experienced recurrence (16 of 20, 80.0%).

**Table 2:** Associations between *SLC39A6* rs1050631 genotypes and recurrence and survival

SNP	Genotype	Recurrence	Unadjusted			Adjusted^a^		
		No(n)/Yes(n)	HR	95%CI	*p*	HR	95%CI	*p*
Total (*n* = 512)		178/334						
rs1050631	CC	139/214	1(reference)			1(reference)		
	CT	35/104	1.813	1.09–3.01	**0.022**	1.379	1.090–1.744	**0.007**
	TT	4/16	1.400	1.11–1.77	**0.005**	1.441	0.864–2.403	0.161
	CT + TT	39/120	1.440	1.15–1.80	**0.001**	1.387	1.108–1.735	**0.004**
SNP	Genotype	Survival	Unadjusted			Adjusteda		
		Ali(n)/Dea(n)	HR	95%CI	*p*	HR	95%CI	*p*
Total(*n* = 512)		182/330						
rs1050631	CC	143/210	1(reference)			1(reference)		
	CT	35/104	1.440	1.138–1.823	**0.002**	1.416	1.119–1.792	**0.004**
	TT	4/16	1.947	1.170–3.239	**0.010**	1.523	0.912–2.542	0.107
	CT + TT	39/120	1.492	1.192–1.868	**< 0.001**	1.429	1.141–1.790	**0.002**

The bold values indicate *p* value < 0.05

*Abbreviations: HR* hazard ratio, *CI* Confidence Interval

^a^Data were calculated using multivariable Cox models that adjusted for gender, age and lymph node metastasis

Univariable analysis showed risk of recurrence to be markedly higher in patients with the CT genotype (HR 1.813, *p* = 0.022) or the CT + TT genotype (HR 1.440, *p* = 0.001) (Table 2). Similar results were obtained with multivariable analysis: CT genotype, HR 1.379, *p* = 0.007; CT + TT genotype, HR 1.387, *p* = 0.004. In order to isolate factors that may influence post-resection outcomes in patients with GA, we stratified our patients by sex, age, tumor size, histologic grade,postoperative chemotherapy status and lymph node metastasis. Multivariable analysis showed that patients with the CT genotype were at increased recurrence risk if they were male (HR 1.564, *p* = 0.001), aged ≥60 years (HR 1.512, *p* = 0.009), with a tumor ≥5 cm (HR 1.807, *p* = 0.001) or with a moderately differentiated tumor (HR 1.830, *p* < 0.001) than those patients with CC genotype had (Additional file 1: Table S1). Similar results were obtained for patients with the or CT + TT genotype: male, HR 1.545, *p* = 0.001; age, HR 1.529, *p* = 0.005; tumor ≥5 cm, HR 1.789, *p* = 0.001; and moderately differentiated tumor, HR 1.780, *p* < 0.001 (Additional file 1: Table S1). In terms of the associations among the genotypes and recurrence risk involved chemotherapy status, Multivariable analysis exhibited that patients with CT, TT or CT + TT had higher recurrence risk only appeared in the group of patients without performing postoperative chemotherapy (Additional file 1: Table S1).

**Fig. 1:**
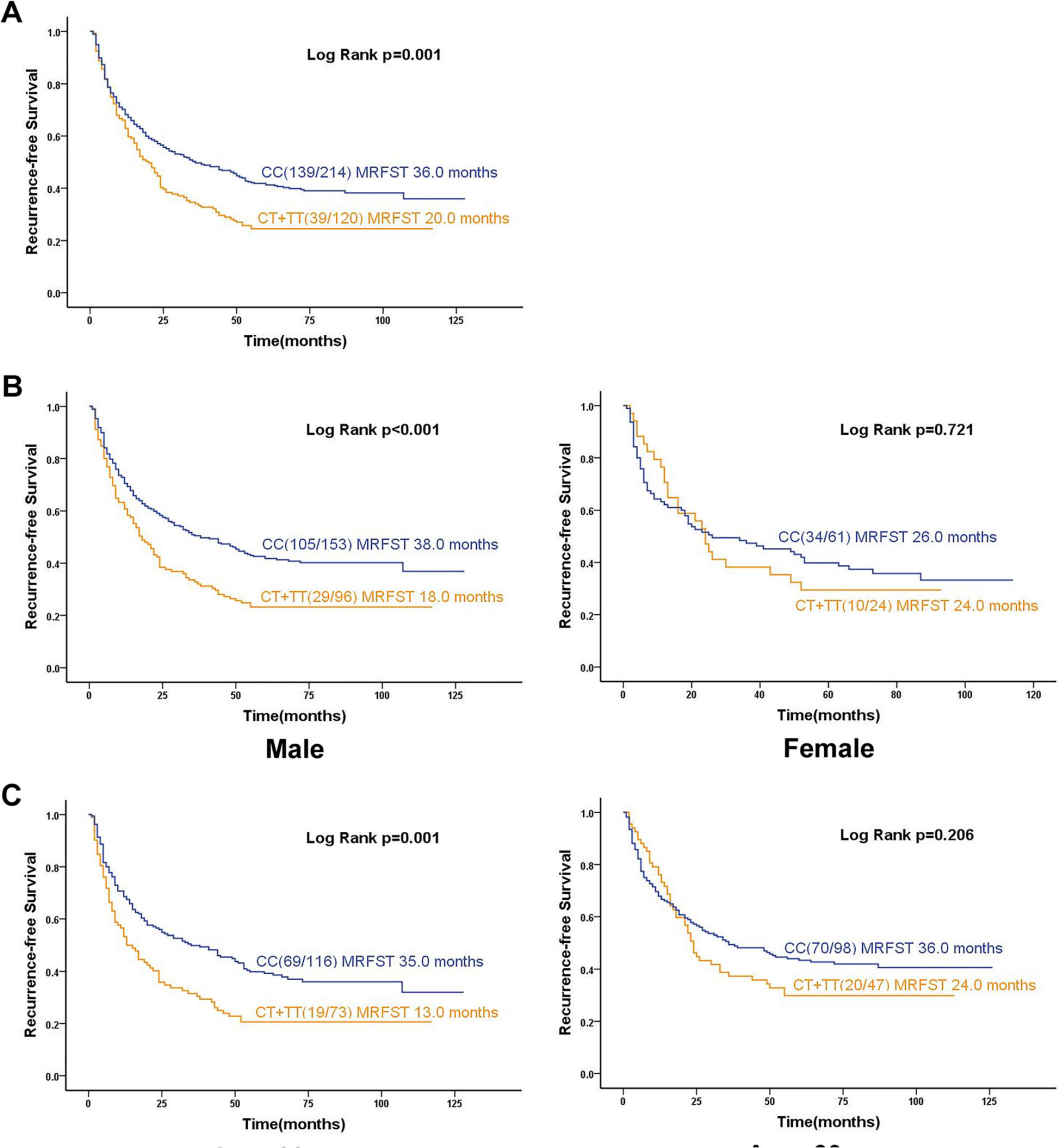
Kaplan–Meier curves of median recurrence-free survival time (MRFST) based on *SLC39A6* rs1050631 genotype. **a** Curves calculated for the whole cohort. **b**-**c** Curves calculated for subgroups stratified by **b** sex and **c** age. The log-rank test was used to calculate *p* values

**Fig. 2:**
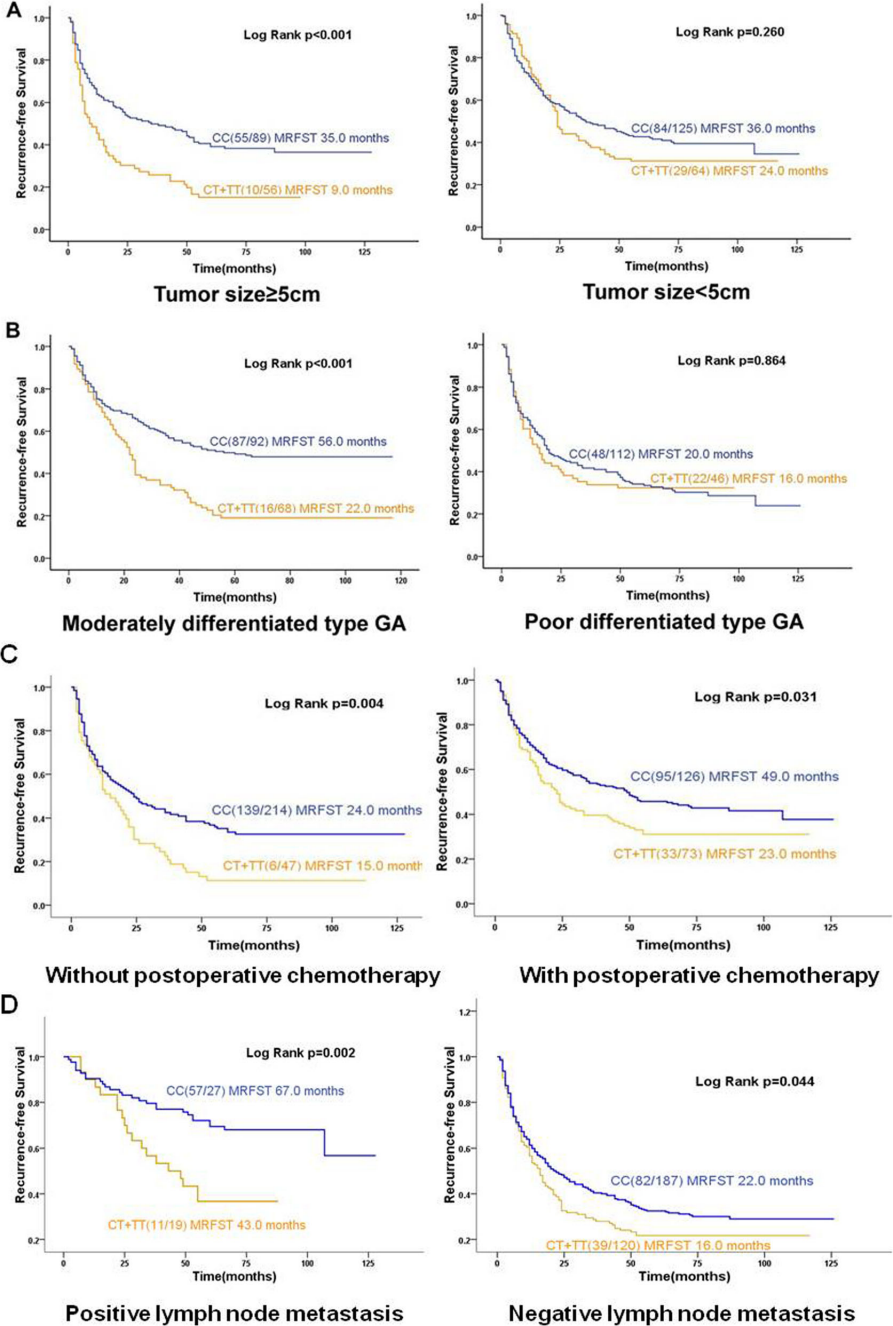
Subgroup Kaplan–Meier curves of median recurrence-free survival time (MRFST) based on *SLC39A6* rs1050631 genotype. Curves calculated for subgroups stratified by **a** tumor size, **b** differentiation grade, **c** chemotherapy status and **d** lymph node metastasis status. The log-rank test was used to calculate *p* values

Kaplan–Meier and log-rank analyses showed that median recurrence-free survival time was only 20 months in patients with the CT + TT genotype, significantly shorter than the 36 months in patients with the CC genotype (*p* = 0.001, Fig. 1a). Stratification analyses based on sex, age, differentiation grade, tumor size, histologic grade and postoperative chemotherapy status showed that patients with the CT + TT genotype who were male, aged ≥60 years or who had a tumor ≥5 cm or had a moderately differentiated tumor or no matter patients who whether performed postoperative chemotherapy had significantly shorter median recurrence-free survival time than patients with the CC genotype had (sex: 18 vs. 38 months, *p* < 0.001; age, 13 vs. 35 months, *p* = 0.001; tumor size, 9 vs. 35 months, *p* < 0.001; moderately differentiated tumor, 22 vs. 56 months, *p* < 0.001; had performed postoperative chemotherapy: 23 vs. 49 months, *p* = 0.031; or had not perform postoperative chemotherapy: 15 vs. 24 months, *p* = 0.004. Figure 1b, c and Fig. 2).

### Associations between SLC39A6 rs1050631 genotype and overall survival

In the entire cohort of 512 patients, 330 (64.5%) died, similar to the proportion of patients with the CC genotype who died (210 of 353, 59.5%; *p* = 0.125). A significantly higher rate of death occurred among patients with the CT (104 of 139, 74.8%) or TT genotype (16 of 20, 80%), based on the chi-squared test and chi-square partitioning (Table 1).

Univariable Cox analysis revealed markedly increased risk of death in patients with the CT genotype (HR 1.440, *p* = 0.002) and also in patients with the CT + TT genotype (HR 1.492, *p* < 0.001). Similarly, multivariable Cox analysis revealed markedly increased risk of death in patients with the CT genotype (HR 1.416, *p* = 0.004) or the CT + TT genotype (HR 1.429, *p* = 0.002), after adjusting for age, sex, and lymph node metastasis status (Table 2). Stratification by sex showed that risk of death was significantly higher among male patients with the CT genotype (HR 1.601, *p* = 0.001) or CT + TT genotype (HR 1.586, *p* < 0.001) than among the patients with CC genotype (Additional file 1: Table S2). Similarly, stratification by age, tumor size, differentiation grade, postoperative chemotherapy status or lymph node metastasis revealed significantly increased risk of death among patients with the CT or CT + TT genotype when they were aged ≥60 years (HR 1.531, *p* = 0.007; HR 1.542, *p* = 0.004) or had a tumor ≥5 cm (HR 1.928, *p* < 0.001; HR 1.904, *p* < 0.001) or a moderately differentiated GA (HR 1.735, *p* = 0.001; HR 1.706, *p* = 0.001) (Additional file 1: Table S2). In terms of the associations among the genotypes and death risk involved chemotherapy status, no matter patients had or had not performed postoperative chemotherapy, who carried CT or CT + TT genotypes showed higher death risk than those carried CC genotype (Additional file 1: Table S2).

**Fig. 3:**
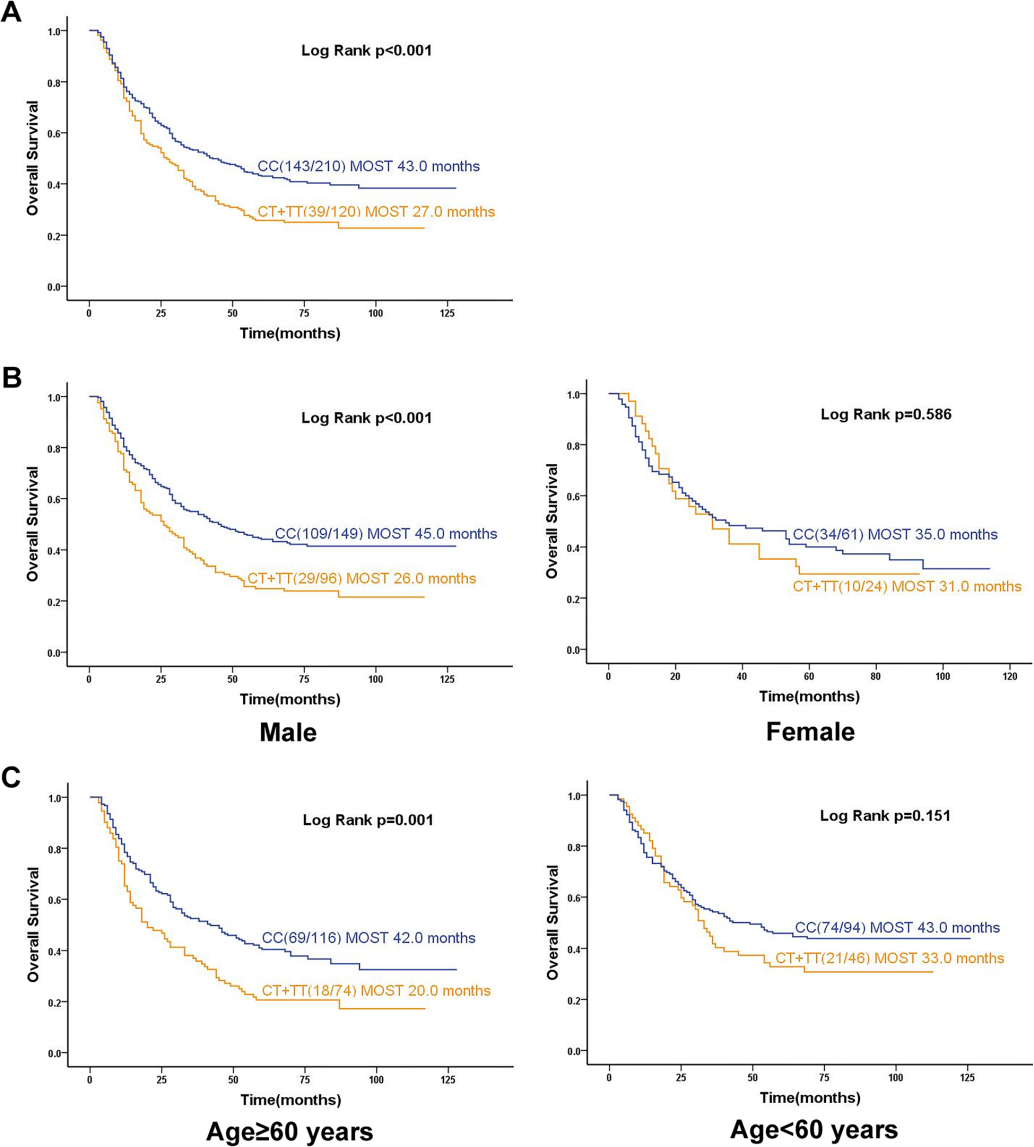
Kaplan–Meier curves of median overall survival time (MOST) based on *SLC39A6* rs1050631 genotype. **a** Curves calculated for the whole cohort. **b-c** Curves were calculated for subgroups stratified by **b** sex and **c** age. The log-rank test was used to calculate *p* values

**Fig. 4:**
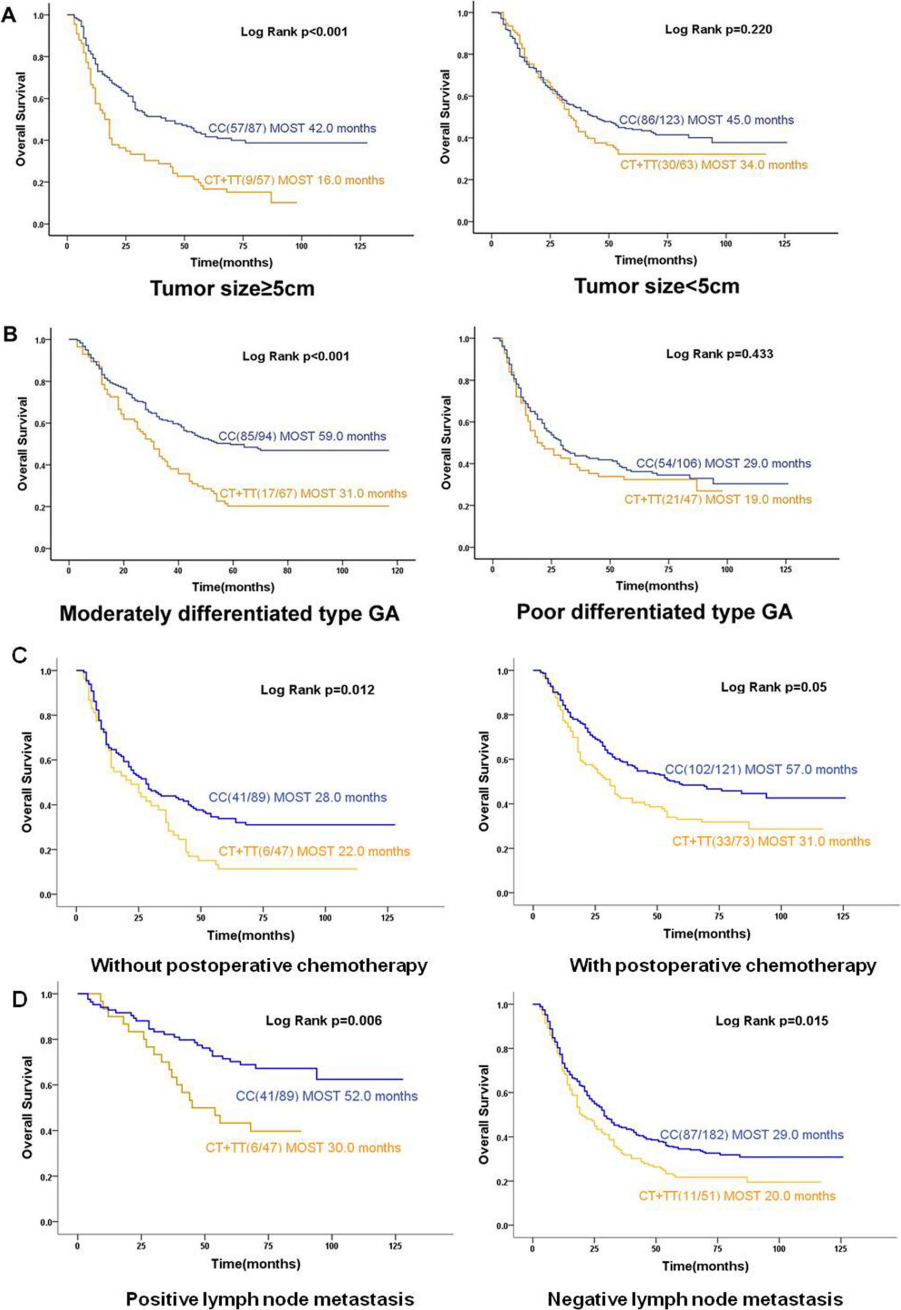
Subgroup Kaplan–Meier curves of median overall survival time (MOST) based on *SLC39A6* rs1050631 genotype. Curves were calculated for subgroups stratified by **a** tumor size, **b** differentiation grade, **c** chemotherapy status and **d** lymph node metastasis status. The log-rank test was used to calculate *p* values

Patients with the CC genotype showed median overall survival time of 43 months, compared to 27 months for patients with the CT + TT genotype (*p* < 0.001; Fig. 3a). Stratification analyses showed that survival time was significantly shorter in patients with the CT + TT genotype if they had any of the following characteristics: male (26 vs. 45 months, *p* < 0.001), aged ≥60 years (20 vs. 42.0 months, *p* = 0.001), had a tumor ≥5 cm (16 vs. 42 months, *p* < 0.001), had moderately differentiated GA (31 vs. 59 months, *p* < 0.001) or no matter patients who whether performed postoperative chemotherapy: had performed postoperative chemotherapy: 31 vs. 57 months, *p* = 0.05, which almost equal to have statistically significant difference; or had not perform postoperative chemotherapy: 22 vs. 28 months, *p* = 0.012.(Fig. 3b, c and Fig. 4).

### SLC39A6 overexpression in GA

**Fig. 5:**
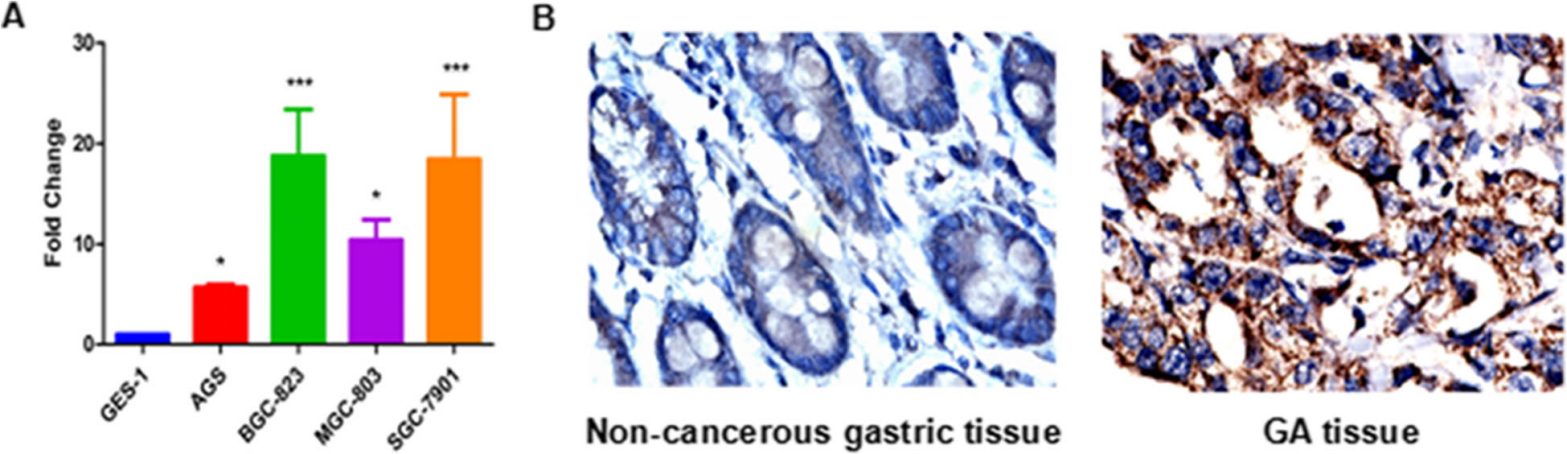
SLC39A6 expression in gastric cancer cell lines and tissues. **a** Quantitative RT-PCR showed that SLC39A6 mRNA levels were significantly higher in the gastric cancer cell lines AGS, BGC-823, SGC-7901 and MGC-803 than in normal gastric cell line GES-1 (all *p* < 0.05). **b** Immunohistochemistry of tissue slices showing up-regulation of SLC39A6 protein in gastric adenocarcinoma (GA) tissues relative to non-cancerous gastric tissue. Magnification, × 400

The results of the above mentioned experiments suggest an association between *SLC39A6* rs1050631 and post-resection outcomes in patients with GA. This raised the question of whether SLC39A6 expression might be associated with GA. We used quantitative PCR and immunohistochemistry to assess expression levels in four GA cell lines (AGS, BGC-823, SGC-7901 and MGC-803) and our cohort of GA tissues. We found that the protein was overexpressed in all four cell lines relative to the normal gastric cell line GES-1, and that the protein was present (immunopositivity scores of ≥1+) in 150 of 198 (75.76%) GA tissues, compared to only 48 of 83 (57.83%) non-cancerous gastric tissues (*p* = 0.003; Fig. 5a and b).

### SLC39A6 knockdown inhibits proliferation, migration and invasion of GA cells

**Fig. 6:**
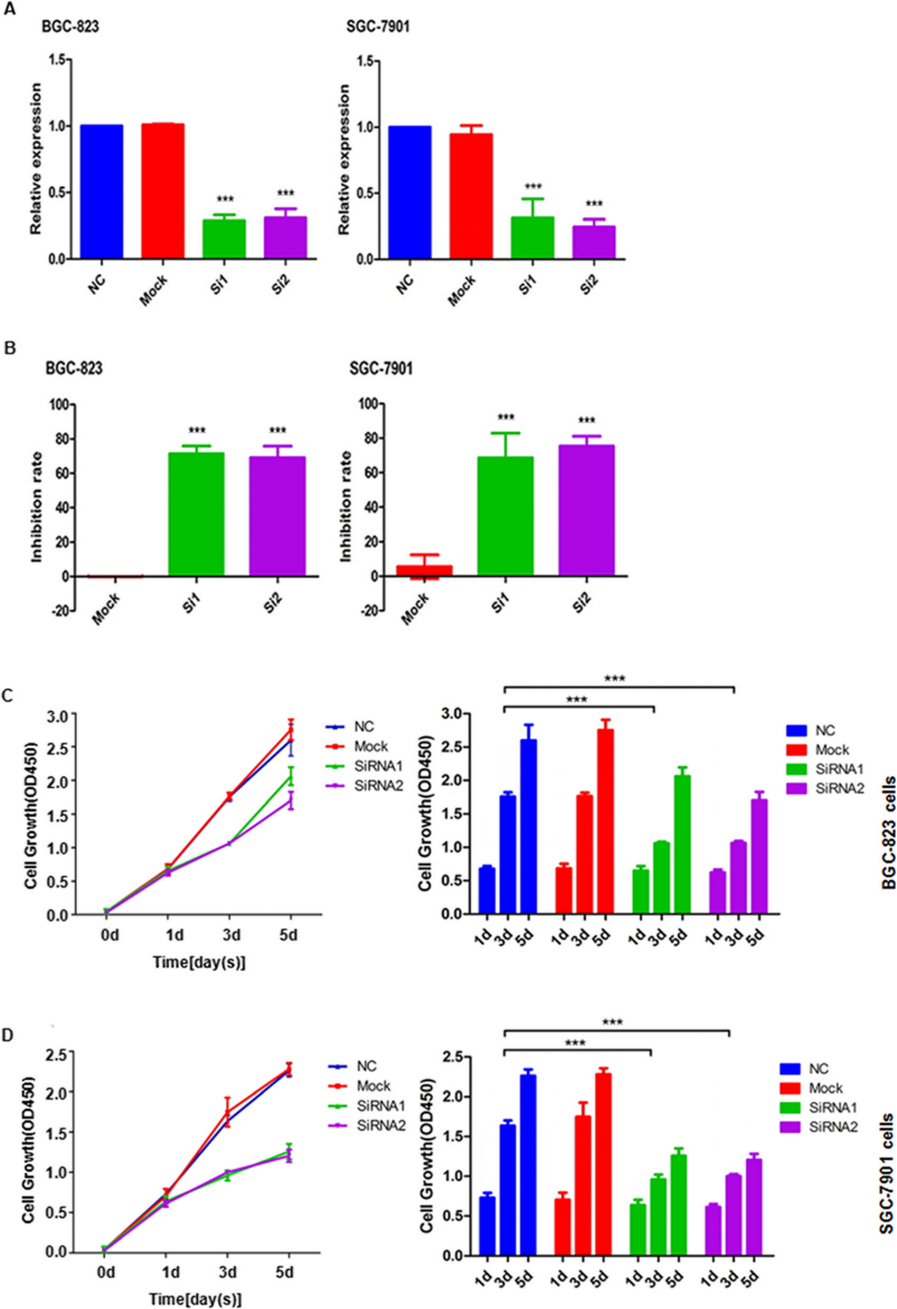
SLC39A6 silencing significantly decreases gastric cancer cell proliferation. **a-b** Quantitative RT-PCR shows that transfection of gastric cancer cell lines BGC-823 and SGC-7901 with SLC39A6 siRNA1 and siRNA2 significantly suppressed SLC39A6 expression. **a** Levels of SLC39A6 mRNA. **b** Extent of SLC39A6 knockdown achieved with each siRNA. **c**-**d** SLC39A6 knockdown inhibited proliferation of **c** BGC-823 and **d** SGC-7901 cells (*p* < 0.05 vs. control cells untransfected (Mock) or transfected with scrambled siRNA (NC))

**Fig. 7:**
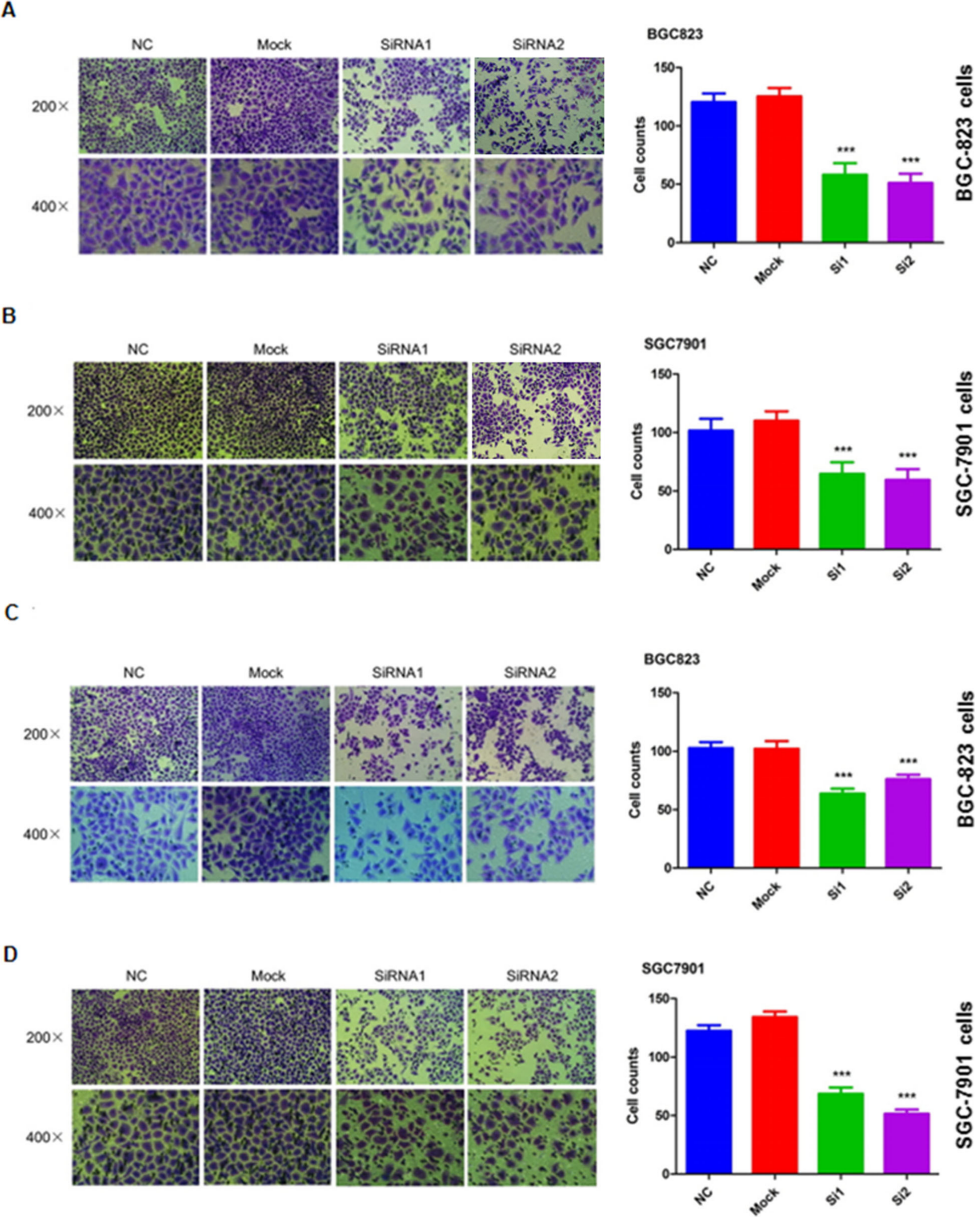
SLC39A6 silencing significantly decreases gastric cancer cell migration and invasion. **a-b** SLC39A6 knockdown inhibited migration by **a** BGC-823 and **b** SGC-7901 cells (*p* < 0.05 vs. Mock or NC controls). **c-d** SLC39A6 knockdown inhibited invasion by **c** BGC-823 and **d** SGC-7901 cells

We designed two short interfering siRNAs to inhibit SLC39A6 expression in the GA cell lines BGC-823 and SGC-7901 (Fig. 6a and b), and this knockdown led to significantly less proliferation in both lines than in untransfected cells (Mock) or cells transfected with scrambled control siRNA (Fig. 6c and d). Knockdown also significantly reduced migration (Fig. 7a and b) and invasion by both cell lines (Fig. 7c and d). These results suggest that *SLC39A6* may contribute to GA by functioning as a typical oncogene.

### Association of different genotype at SLC39A6 rs1050631 with SLC39A6 expression in GA

**Fig. 8:**
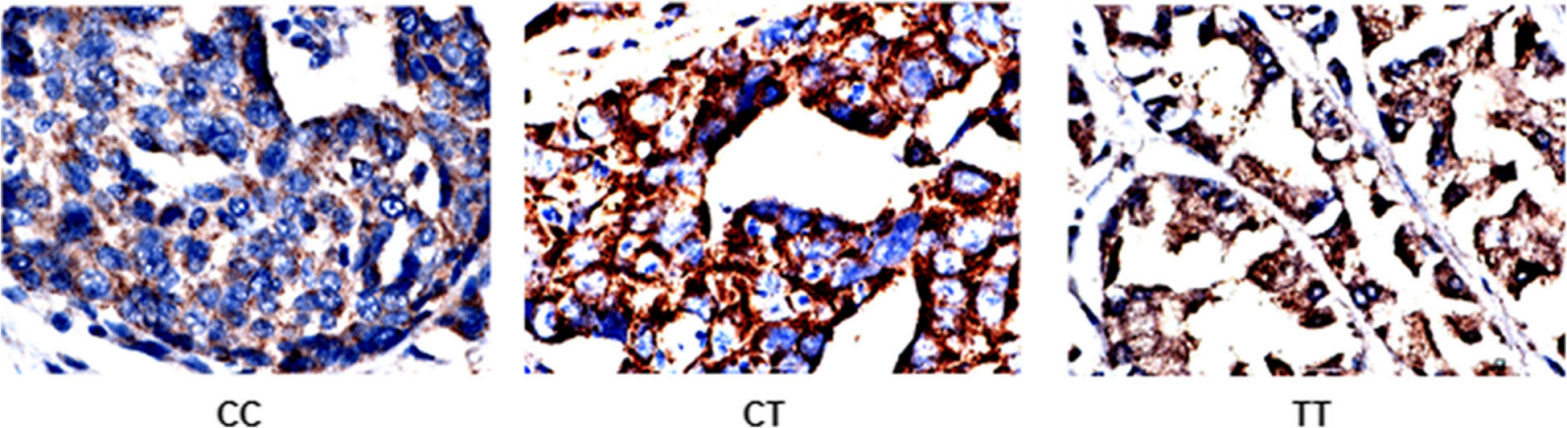
Representative immunohistochemistry results of SLC39A6 protein in gastric cancer tissue from patients with different genotypes (CC, CT, TT) at *SLC39A6* rs1050631. Magnification, × 400

Immunohistochemistry of 198 GA tissues from our patient cohort detected high SLC39A6 protein expression (≥2+) in 86 tissues (43.43%; Fig. 8); high expression was detected in 6 of 14 (42.86%) patients with TT, 50 of 92 (54.35%) patients with CT, 56 of 106 (52.83%) patients with CT + TT, and 31 of 92 (33.70%) patients with CC genotype. Though the high positive expression rate among the three different genotypes groups showed no statistically significantly different from that in the whole group (*p* > 0.05), the rate of high SLC39A6 expression was significantly greater among patients with CT genotype than among those with CC genotype (*p* = 0.005) based on chi-square partitioning (Table 3). We speculate that the *SLC39A6* rs1053631 genotype is associated with post-resection prognosis of patients with GA because the CT genotype leads to higher SLC39A6 expression.

**Table 3:** Chi-square partitioning to test the association between *SLC39A6* rs1050631 and SLC39A6 expression detected by immunohistochemistry

SNP	Genotype	expression of SLC39A6		Case-total^a^	Case-case^b^
		Low(n)/ High(n)	High expression rate (%)	*p* < 0.00833	*p* < 0.0125
Total(*n* = 198)		112/86	43.4		
rs1050631	CC	61/31	33.7	0.116	1vs2:0.005
	CT	42/50	54.3	0.083	2vs3:0.422
	TT	8/6	42.9	0.966	1vs3:0.712

^a^Case–total: *p* value for the comparison between the group with the indicated genotype and the total group of 198 samples;

^b^Case–case: *p* value for the pairwise comparisons between groups with different genotypes

## Discussion

Occurrence and prognosis of gastric cancer are influenced by genetics, lifestyle and the environment [20, 21]. Single-nucleotide polymorphisms in certain genes may alter the levels or activity of proteins in a way that leads to cancer [22]. Here we analyzed the potential association of a particular polymorphism with GA and post-resection recurrence and death in a population living in a part of China with one of the highest incidences of gastric cancer in this country. This population may contain genetic elements that predispose them to GA, offering unique opportunities for studying genetic risk factors and prognostic markers, especially since most genetic risk studies are not conducted in high-incidence areas. Our data may also strengthen our understanding of risk factors in a high-risk environment.

Although several polymorphisms have been linked to gastric cancer, we focused on polymorphism in the *SLC39A6 (LIV-1)* gene because it is a downstream target of the STAT3 oncoprotein and promotes cancer progression by increasing cell growth and differentiation [23]. SLC39A6 can influence the epithelial–mesenchymal transition in pancreatic cancer cells, making their phenotype more aggressive [24]; and treatments that target SLC39A6 can reduce metastasis and predict prognosis of patients with hepatocellular carcinoma [25], and it can inhibit progression of metastatic breast cancer [26]. In particular, we focused on the *SLC39A6* rs1050631 polymorphism. This polymorphism shows a strong association with death risk of ESCC in extensive studies involving more than a thousand of Chinese from at least two different parts of the country [14]. We are unaware of reports exploring a possible association between this polymorphism and GA.

Our study suggests that GA patients with the CT + TT genotype at rs1050631 are at significantly higher risk of recurrence and death than patients with CC genotype. Male gender, age older than 60 years, having a tumor ≥5 cm or having a moderately differentiated tumor are associated with worse prognosis in patients with the CT + TT genotype than in those with the CC genotype. It is still unknown the reason why *SLC39A6* rs1050631 polymorphism had different effect on affecting the recurrence risk and death risk in GA patients with and without performing postoperative chemotherapy. Patients with CT or CT + TT genotypes were only associated with increased recurrence risk in the group of patients no carrying out postoperative chemotherapy. Whereas, patients with CT or CT + TT genotypes were associated with increased death risk both in the groups of patients carry and no carrying out postoperative chemotherapy. However, patients with CT + TT genotypes had both significantly decreased recurrence-free survival time and overall survival time than those with CC genotype. About these, it needs further investigation to reveal the reasons.

This observation of an association between *SLC39A6* rs1050631 and post-resection outcomes in patients with GA prompted to ask whether SLC39A6 expression is associated with GA. Therefore we compared expression levels in a panel of GA cell lines as well as in our cohort of GA patients. The results show an association between SLC39A6 up-regulation and GA. We further found that the CT + TT genotype at *SLC39A6* rs1050631, which was associated with worse clinical outcomes in our cohort. Consistent with a role of *SLC39A6* in GA, we found that knocking down the protein in GA cell lines inhibited their proliferation, migration and invasion. These results illustrating SLC39A6 overexpression in GA tissues and GA cell lines as well as anti-cancer effects of SLC39A6 down-regulation mirror results published for SLC39A6 in ESCC [14]*.* These findings indicate that, as in ESCC and several other cancers, SLC39A6 appears to participate in GA, such that polymorphisms affecting its expression may be useful prognostic markers. Our results further suggest a model in which *SLC39A6* rs1050631 alters the expression of SLC39A6 and thereby influences post-resection outcomes.

We identified several clinico-demographic characteristics associated with worse post-resection outcomes, including male gender, older age and having larger, moderately differentiated tumors or chemotherapy status.

## Conclusion

In summary, our study provides preliminary evidence that SLC39A6 is involved in GA, and that genotype at *SLC39A6* rs1050631 can predict post-resection prognosis of GA patients, at least in a population in an area with high GA incidence. Although we focused here on *SLC39A6* rs1050631 because of the strong evidence in favor of a linkage with ESCC [14], future research is needed to explore other single-nucleotide polymorphisms that may be involved in gastric cancer. Due to this study has its limitation: all the clinical samples were obtained from single center in Fujian Province, further studies are also needed to examine whether our findings in this Chinese population can be generalized to other ethnic groups.

## Supplementary information

**Additional file 1: Table S1.**Associations between SLC39A6 rs1050631 genotypes and recurrent after stratification by sex, age, tumor size, differentiation grade,chemotherapy status and lymph node metastasis. **Table S2.** Associations between SLC39A6 rs1050631 genotypes with survival, after stratification by sex, age, tumor size, differentiation grade,chemotherapy status and lymph node metastasis.

## Data Availability

The datasets used and/or analyzed during the current study are available from the corresponding author on reasonable request.

## Abbreviations

ESCC: Esophageal squamous cell carcinoma
GA: Gastric adenocarcima
HR: Hazard ratios;CI:confidence intervals
NC: Negative control
TNM: Tumor-node-metastasis
