# Supplementary material for: Involvement of SLC39A6 in gastric adenocarcinoma and correlation of the SLC39A6 polymorphism rs1050631 with clinical outcomes after resection
Source: BMC Cancer. 2019 Nov 8;19:1069. doi: 10.1186/s12885-019-6222-z (PMC6839152; doi:10.1186/s12885-019-6222-z)
Supplement: Supplementary file 1 — Additional file 1: Table S1.Associations between SLC39A6 rs1050631 genotypes and recurrent after stratification by sex, age, tumor size, differentiation grade,chemotherapy status and lymph node metastasis. Table S2. Associations between SLC39A6 rs1050631 genotypes with survival, after stratification by sex, age, tumor size, differentiation grade,chemotherapy status and lymph node metastasis. [file 12885_2019_6222_MOESM1_ESM.docx]

Table S1. Associations between *SLC39A6* rs1050631 genotypes and recurrence, after stratification by sex, age, tumor size, differentiation grade, chemotherapy status or lymph node metastasis.

| SNP | Genotype | Gender | | | | | | | |
| --- | --- | --- | --- | --- | --- | --- | --- | --- | --- |
|  |  | Male | | | | Female | | | |
|  |  | Recurrence | | | | Recurrence | | | |
|  |  | No(n)/Yes(n) | HR | 95%CI | *p^a^* | No(n)/Yes(n) | HR | 95%CI | *p^a^* |
| Total  (n=512) |  | 134/249 |  |  |  | 44/85 |  |  |  |
|  | CC | 105/153 | 1(reference) | | | 34/61 | 1(reference) | | |
|  | CT | 26/84 | 1.564 | 1.197-2.044 | 0.001 | 9/20 | 0.951 | 0.572-1.581 | 0.846 |
|  | TT | 3/12 | 1.424 | 0.788-2.573 | 0.241 | 1/4 | 1.456 | 0.524-4.048 | 0.471 |
|  | CT+TT | 29/96 | 1.545 | 1.196-1.996 | 0.001 | 10/24 | 1.008 | 0.626-1.624 | 0.973 |
| SNP | Genotype | Age | | | | | | | |
|  |  | <60 | | | | ≥60 | | | |
|  |  | Recurrence | | | | Recurrence | | | |
|  |  | No(n)/Yes(n) | HR | 95%CI | *p^b^* | No(n)/Yes(n) | HR | 95%CI | *p^b^* |
| Total  (n=512) |  | 90/145 |  |  |  | 88/189 |  |  |  |
|  | CC | 70/98 | 1(reference) | | | 69/116 | 1(reference) | | |
|  | CT | 18/43 | 1.242 | 0.866-1.782 | 0.238 | 17/61 | 1.512 | 1.108-2.065 | 0.009 |
|  | TT | 2/4 | 1.127 | 0.414-3.071 | 0.814 | 2/12 | 1.620 | 0.890-2.948 | 0.115 |
|  | CT+TT | 20/47 | 1.232 | 0.868-1.747 | 0.243 | 19/73 | 1.529 | 1.139-2.052 | 0.005 |
| SNP | Genotype | Tumor Size | | | | | | | |
|  |  | <5cm | | | | ≥5cm | | | |
|  |  | Recurrence | | | | Recurrence | | | |
|  |  | No(n)/Yes(n) | HR | 95%CI | *p^c^* | No(n)/Yes(n) | HR | 95%CI | *p^c^* |
| Total  (n=512) |  | 113/189 |  |  |  | 65/145 |  |  |  |
|  | CC | 84/125 | 1(reference) | | | 55/89 | 1(reference) | | |
|  | CT | 27/54 | 1.131 | 0.820-1.558 | 0.453 | 8/50 | 1.807 | 1.269-2.575 | 0.001 |
|  | TT | 2/10 | 1.333 | 0.696-2.554 | 0.386 | 2/6 | 1.657 | 0.720-3.811 | 0.235 |
|  | CT+TT | 29/64 | 1.158 | 0.856-1.567 | 0.341 | 10/56 | 1.789 | 1.273-2.516 | 0.001 |

| SNP | Genotype | Histologic grade | | | | | | | | | | | | |
| --- | --- | --- | --- | --- | --- | --- | --- | --- | --- | --- | --- | --- | --- | --- |
|  |  | Poorly differentiated | | | | Moderately differentiated | | | | Well differentiated | | | | |
|  |  | Recurrence | | | | Recurrence | | | | Recurrence | | | | |
|  |  | No(n)/Yes(n) | HR | 95%CI | *p^c^* | No(n)/  Yes(n) | HR | 95%CI | *p^c^* | No(n)/Yes(n) | HR | 95%CI | | *p^c^* |
| Total  (n=512) |  | 70/158 |  | | | 103/160 |  | | | 5/16 |  | | | |
|  | CC | 48/112 | 1(reference) | | | 87/92 | 1(reference) | | | 4/10 | 1(referrence) | | | |
|  | CT | 20/38 | 1.052 | 0.73-1.52 | 0.789 | 14/61 | 1.830 | 1.32-2.54 | <0.001 | 1/5 | 1.827 | 0.54-6.22 | 0.335 | |
|  | TT | 2/8 | 1.468 | 0.71-3.03 | 0.298 | 2/7 | 1.421 | 0.65-3.10 | 0.376 | 0/1 | NA^d^ | NA^d^ | NA^d^ | |
|  | CT+TT | 22/46 | 1.107 | 0.78-1.56 | 0.565 | 16/68 | 1.780 | 1.29-2.45 | <0.001 | 1/6 | 2.131 | 0.67-6.78 | 0.200 | |

| SNP | Genotype | Chemotherapy |  | | | | | | |
| --- | --- | --- | --- | --- | --- | --- | --- | --- | --- |
|  |  | Yes | | | | No | | | |
|  |  | Recurrence | | | | Recurrence | | | |
|  |  | No(n)/Yes(n) | HR | 95%CI | *p^c^* | No(n)/Yes(n) | HR | 95%CI | *p^c^* |
| Total |  | 128/201 |  |  |  | 50/131 |  |  |  |
| (n=512) | CC | 95/128 | 1(reference) |  |  | 44/84 | 1(reference) |  |  |
|  | CT | 29/64 | 1.27 | 0.94-1.72 | 0.981 | 6/40 | 1.79 | 1..21-2.63 | 0.003 |
|  | TT | 4/9 | 1.01 | 0.51-2.01 | 0.571 | 0/7 | 2.78 | 1.26-6.13 | 0.012 |
|  | CT+TT | 33/73 | 1.24 | 0.92-1.65 | 0.153 | 6/47 | 1.89 | 1.30-2.73 | <0.001 |
| SNP | Genotype | Lymph node metastasis | | | | | | | |
|  |  | Yes | | | | No | | | |
|  |  | Recurrence | | | | Recurrence | | | |
|  |  | No(n)/Yes(n) | HR | 95%CI | *P^e^* | No(n)/Yes(n) | HR | 95%CI | *P^e^* |
| Total |  | 110/288 |  |  |  | 68/46 |  |  |  |
| (n=512) | CC | 82/187 | 1(reference) |  |  | 57/27 | 1(reference) |  |  |
|  | CT | 24/86 | 1.33 | 0.79-2.26 | 0.289 | 11/18 | 13.65 | 1.69-110.56 | 0.014 |
|  | TT | 4/15 | 1.26 | 0.98-1.63 | 0.075 | 0/1 | 2.48 | 1.35-4.55 | 0.003 |
|  | CT+TT | 28/101 | 0.89 | 0.79-1.00 | 0.153 | 11/19 | 0.62 | 0.46-0.84 | 0.002 |

The bold values indicate p value < 0.05.

^a^Data were calculated using a multivariable Cox model that adjusted for age and lymph node metastasis.

^b^Data were calculated using a multivariable Cox model that adjusted for gender and lymph node metastasis.

^c^Data were calculated using a multivariable Cox model that adjusted for gender, age and lymph node metastasis.

^d^Data were not available.

^e^Data were calculated using a multivariable Cox model that adjusted for gender and age.

Abbreviations: HR, hazard ratio; CI, Confidence Interval.

Table S2.Associations between *SLC39A6* rs1050631 genotypes with survival, after stratification by sex, age, tumor size, differentiation grade,chemotherapy status or lymph node metastasis.

| SNP | Genotype | Gender | | | | | | | | | |
| --- | --- | --- | --- | --- | --- | --- | --- | --- | --- | --- | --- |
|  |  | Male | | | | | Female | | | | |
|  |  | Ali(n)/Dea(n) | HR | | 95%CI | *p^a^* | Ali(n)/Dea(n) | HR | | 95%CI | *p^a^* |
| Total  (n=512) |  | 138/245 |  | |  |  | 44/85 |  | |  |  |
|  | CC | 109/149 | 1(reference) | | | | 34/61 | 1(reference) | | | |
|  | CT | 26/84 | 1.601 | | 1.224-2.093 | 0.001 | 9/20 | 0.985 | 0.592-1.640 | | 0.954 |
|  | TT | 3/12 | 1.486 | | 0.822-2.688 | 0.190 | 1/4 | 1.654 | 0.595-4.600 | | 0.335 |
|  | CT+TT | 29/96 | 1.586 | | 1.226-2.051 | <0.001 | 10/24 | 1.055 | 0.654-1.701 | | 0.826 |
| SNP | Genotype | Age | | | | | | | | | |
|  |  | <60 | | | | | ≥60 | | | | |
|  |  | Ali(n)/Dea(n) | | HR | 95%CI | *p^b^* | Ali(n)/Dea(n) | HR | | 95%CI | *p^b^* |
| Total  (n=512) |  | 95/140 | |  |  |  | 87/190 |  | |  |  |
|  | CC | 74/94 | | 1(reference) | | | 69/116 | 1(reference) | | | |
|  | CT | 19/42 | | 1.282 | 0.889-1.847 | 0.183 | 16/62 | 1.531 | | 1.123-2.087 | 0.007 |
|  | TT | 2/4 | | 1.290 | 0.473-3.517 | 0.618 | 2/12 | 1.605 | | 0.880-2.926 | 0.123 |
|  | CT+TT | 21/46 | | 1.282 | 0.900-1.827 | 0.169 | 18/74 | 1.542 | | 1.150-2.068 | 0.004 |
| SNP | Genotype | Tumor Size | | | | | | | | | |
|  |  | <5cm | | | | | ≥5cm | | | | |
|  |  | Ali(n)/Dea(n) | | HR | 95%CI | *p^c^* | Ali(n)/Dea(n) | HR | | 95%CI | *p^c^* |
| Total  (n=512) |  | 116/186 | |  |  |  | 66/144 |  | |  |  |
|  | CC | 86/123 | | 1(reference) | | | 57/87 | 1(reference) | | | |
|  | CT | 28/53 | | 1.141 | 0.826-1.577 | 0.423 | 7/51 | 1.928 | | 1.355-2.742 | <0.001 |
|  | TT | 2/10 | | 1.433 | 0.747-2.749 | 0.279 | 2/6 | 1.726 | | 0.750-3.974 | 0.200 |
|  | CT+TT | 30/63 | | 1.179 | 0.869-1.600 | 0.289 | 9/57 | 1.904 | | 1.355-2.675 | <0.001 |

| SNP | Genotype | | Histologic grade | | | | | | | | | | | | | | |
| --- | --- | --- | --- | --- | --- | --- | --- | --- | --- | --- | --- | --- | --- | --- | --- | --- | --- |
|  |  | | Poorly differentiated | | | | | Moderately differentiated | | | | | Well differentiated | | | | |
|  |  | | Ali(n)/Dea(n) | | HR | 95%CI | *p^c^* | | Ali(n)/Dea(n) | HR | 95%CI | *p^c^* | | Ali(n)/Dea(n) | HR | 95%CI | *p^c^* |
| Total  (n=512) |  | | 75/153 | |  |  |  | | 102/161 |  |  |  | | 5/16 |  |  |  |
|  | | CC | | 54/106 | 1(reference) | | | | 85/94 | 1(reference) | | | | 4/10 | 1(referrence) | | |
|  |  | CT | | 19/39 | 1.209 | 0.84-1.75 | 0.315 | | 15/60 | 1.735 | 1.25-2.41 | 0.001 | | 1/5 | 1.731 | 0.48-6.26 | 0.403 |
|  |  | TT | | 2/8 | 1.479 | 0.72-3.06 | 0.291 | | 2/7 | 1.482 | 0.68-3.23 | 0.322 | | 0/1 | NA^d^ | NA^d^ | NA^d^ |
|  |  | CT+TT | | 21/47 | 1.248 | 0.88-1.76 | 0.209 | | 17/67 | 1.706 | 1.24-2.35 | 0.001 | | 1/6 | 2.192 | 0.66-7.34 | 0.203 |

| SNP | Genotype | Chemotherapy | | | | | | | |
| --- | --- | --- | --- | --- | --- | --- | --- | --- | --- |
|  |  | Yes | | | | No | | | |
|  |  | Ali(n)/Dea(n) | HR | 95%CI | *p^c^* | Ali(n)/Dea(n) | HR | 95%CI | *p^c^* |
| Total  (n=512) |  | 135/194 |  |  |  | 47/136 |  |  |  |
|  | CC | 102/121 | 1(reference) | | | 41/89 | 1(reference) | | |
|  | CT | 29/64 | 1.40 | 1.03-1.89 | 0.032 | 6/40 | 1.66 | 1.13-2.43 | 0.010 |
|  | TT | 4/9 | 1.40 | 0.58-2.30 | 0.693 | 0/7 | 2.47 | 1.12-5.46 | 0.025 |
|  | CT+TT | 33/73 | 1.36 | 1.01-1.83 | 0.040 | 6/47 | 1.74 | 1.20-2.53 | 0.004 |
| SNP | Genotype | Lymph node metastasis | | | | | | | |
|  |  | Yes | | | | No | | | |
|  |  | Ali(n)/Dea(n) | HR | 95%CI | *P^e^* | Ali(n)/Dea(n) | HR | 95%CI | *P^e^* |
| Total  (n=512) |  | 114/284 |  |  |  | 47/136 |  |  |  |
|  | CC | 87/182 | 1(reference) | | | 41/89 | 1(reference) | | |
|  | CT | 23/87 | 1.35 | 0.80-2.30 | 0.260 | 6/40 | 1.66 | 1.13-2.43 | 0.010 |
|  | TT | 4/15 | 1.30 | 1.01-1.69 | 0.040 | 0/7 | 2.47 | 1.12-5.46 | 0.025 |
|  | CT+TT | 27/102 | 1.34 | 1.05-1.70 | 0.020 | 6/47 | 2.40 | 1.32-4.39 | 0.004 |

The bold values indicate p value < 0.05.

^a^Data were calculated using a multivariable Cox model that adjusted for age and lymph node metastasis.

^b^Data were calculated using a multivariable Cox model that adjusted for gender and lymph node metastasis.

^c^Data were calculated using a multivariable Cox model that adjusted for gender, age and lymph node metastasis.

^d^Data were not available.

^e^ Data were calculated using a multivariable Cox model that adjusted for gender, age.

Abbreviations: HR, hazard ratio; CI, Confidence Interval.
